# Multimorbidity in patients with heart failure from 11 Asian regions: A prospective cohort study using the ASIAN-HF registry

**DOI:** 10.1371/journal.pmed.1002541

**Published:** 2018-03-27

**Authors:** Jasper Tromp, Wan Ting Tay, Wouter Ouwerkerk, Tiew-Hwa Katherine Teng, Jonathan Yap, Michael R. MacDonald, Kirsten Leineweber, John J. V. McMurray, Michael R. Zile, Inder S. Anand, Carolyn S. P. Lam, ASIAN-HF authors

**Affiliations:** 1 National Heart Centre Singapore, Singapore, Singapore; 2 Department of Cardiology, University Medical Center Groningen, Groningen, Netherlands; 3 Department of Clinical Epidemiology, Biostatistics and Bioinformatics, Academic Medical Center, Amsterdam, Netherlands; 4 School of Population and Global Health, University of Western Australia, Perth, Western Australia, Australia; 5 Changi General Hospital, Singapore, Singapore; 6 Bayer, Wuppertal, Germany; 7 Institute of Cardiovascular and Medical Sciences, University of Glasgow, Glasgow, United Kingdom; 8 School of Medicine, Dentistry and Nursing, University of Glasgow, Glasgow, United Kingdom; 9 Medical University of South Carolina, Charleston, South Carolina, United States of America; 10 Ralph H. Johnson VA Medical Center, Charleston, South Carolina, United States of America; 11 Minneapolis VA Medical Center, Minneapolis, Minnesota, United States of America; 12 National University Heart Centre, Singapore, Singapore; 13 Duke–NUS Medical School, Singapore, Singapore; University of Oxford, UNITED KINGDOM

## Abstract

**Background:**

Comorbidities are common in patients with heart failure (HF) and complicate treatment and outcomes. We identified patterns of multimorbidity in Asian patients with HF and their association with patients’ quality of life (QoL) and health outcomes.

**Methods and findings:**

We used data on 6,480 patients with chronic HF (1,204 with preserved ejection fraction) enrolled between 1 October 2012 and 6 October 2016 in the Asian Sudden Cardiac Death in Heart Failure (ASIAN-HF) registry. The ASIAN-HF registry is a prospective cohort study, with patients prospectively enrolled from in- and outpatient clinics from 11 Asian regions (Hong Kong, Taiwan, China, Japan, Korea, India, Malaysia, Thailand, Singapore, Indonesia, and Philippines). Latent class analysis was used to identify patterns of multimorbidity. The primary outcome was defined as a composite of all-cause mortality or HF hospitalization within 1 year. To assess differences in QoL, we used the Kansas City Cardiomyopathy Questionnaire. We identified 5 distinct multimorbidity groups: elderly/atrial fibrillation (AF) (*N* = 1,048; oldest, more AF), metabolic (*N* = 1,129; obesity, diabetes, hypertension), young (*N* = 1,759; youngest, low comorbidity rates, non-ischemic etiology), ischemic (*N* = 1,261; ischemic etiology), and lean diabetic (*N* = 1,283; diabetic, hypertensive, low prevalence of obesity, high prevalence of chronic kidney disease). Patients in the lean diabetic group had the worst QoL, more severe signs and symptoms of HF, and the highest rate of the primary combined outcome within 1 year (29% versus 11% in the young group) (*p* for all <0.001). Adjusting for confounders (demographics, New York Heart Association class, and medication) the lean diabetic (hazard ratio [HR] 1.79, 95% CI 1.46–2.22), elderly/AF (HR 1.57, 95% CI 1.26–1.96), ischemic (HR 1.51, 95% CI 1.22–1.88), and metabolic (HR 1.28, 95% CI 1.02–1.60) groups had higher rates of the primary combined outcome compared to the young group. Potential limitations include site selection and participation bias.

**Conclusions:**

Among Asian patients with HF, comorbidities naturally clustered in 5 distinct patterns, each differentially impacting patients’ QoL and health outcomes. These data underscore the importance of studying multimorbidity in HF and the need for more comprehensive approaches in phenotyping patients with HF and multimorbidity.

**Trial registration:**

ClinicalTrials.gov NCT01633398

## Abstract 2

Using data from the ASIAN-HF Registry, Carolyn Lam and colleagues examine multimorbidity patterns in patients with heart failure from 11 Asian countries

## Author summary

**Why was this study done?:**

- The prevalence of multimorbidity (≥2 comorbidities) is increasing among patients with heart failure.
- Multimorbidity can impede survival and complicate treatment.
- Yet, previous studies have investigated single comorbidities in isolation.

**What did the researchers do and find?:**

- Using latent class analysis, we identified patterns of multimorbidity among patients with heart failure from 11 regions in Asia in a prospective cohort study of 6,480 patients with heart failure (1,204 with heart failure and a preserved ejection fraction).
- We identified 5 multimorbidity groups: elderly/atrial fibrillation (old, more atrial fibrillation), metabolic (obese, diabetic, hypertensive), young (younger, low prevalence of comorbidities), ischemic (ischemic etiology), and lean diabetic (diabetic, low prevalence of obesity).
- Multimorbidity groups had distinct geographic distributions across Asia and were associated with changes in cardiac structure and function.
- Overall, the lean diabetic group had the strongest association with a combined outcome of mortality or hospitalization for heart failure.

**What do these findings mean?:**

- Our findings suggest that multimorbidity is highly prevalent in patients with heart failure and is associated with a distinct geographic distribution and adverse outcomes.
- Combined, our data underscore the importance of multimorbidity in patients with heart failure and call for more comprehensive approaches in phenotyping patients with heart failure and multimorbidity.

## Introduction

Multimorbidity, the presence of 2 or more chronic medical conditions in an individual, is highly prevalent in patients with heart failure (HF) [1–3]. Indeed, with aging populations worldwide, patients with age-related multimorbidity are becoming the norm rather than the exception. This is especially so in Asia, with the most rapidly aging populations in the world, where almost two-thirds of patients with HF were found to have multimorbidity [4]. Comorbidities and their treatments may complicate the diagnosis, treatment, and outcomes of patients with HF, affect patient preferences for care, and negatively impact patient outcomes.

Within the HF syndrome, we currently distinguish HF with reduced ejection fraction (HFrEF) from HF with preserved ejection fraction (HFpEF). Early HF trials defined HF using a reduced left ventricular ejection fraction (LVEF) as an entry criterion, leading to the distinction of HFrEF from HFpEF since large trials of medications (e.g., renin-angiotensin-aldosterone system blockers) that showed improved survival in HFrEF later failed to improve outcomes in similar trials for HFpEF [5]. Cardiac structure and function are distinct between the HF groups: patients with HFrEF mostly display left ventricular (LV) eccentric remodeling with systolic dysfunction, whereas patients with HFpEF more often have concentric remodeling with preserved LV pump function but prominent diastolic dysfunction and increased filling pressures [6]. The underlying basis for these differences remains poorly understood and has been postulated to be related to the different comorbidity burdens in these patients [7].

Most prior clinical research has focused on individual comorbidities in isolation and has not studied the burden and patterns of multimorbidity in HF. Understanding how comorbidities cluster in individuals, and the impact of clustering of comorbidities on patient outcomes, is an important step towards personalizing HF treatment approaches for better outcomes [5,8–13].

Thus, we sought to identify the patterns and burden of multimorbidity in Asian patients with HF, as well as the association of specific multimorbidity patterns with patients’ quality of life (QoL), cardiac remodeling, and health outcomes. We hypothesized that comorbidities would cluster in specific multimorbidity groups, regardless of ejection fraction, and that these groups would differentially influence patients’ QoL, cardiac remodeling and health outcomes. Furthermore, we hypothesized that regional variation would exist across Asia, providing important insights for healthcare resource allocation and a tailored approach to patients from different Asian regions.

## Methods

### Study design, study population, and setting

This study is reported as per the Strengthening the Reporting of Observational Studies in Epidemiology (STROBE) guidelines (S1 Checklist). Ethics approvals were obtained from the relevant human ethics committees at all sites. All patients included provided written informed consent, and this study adheres to the principles of medical research as laid down in the Declaration of Helsinki. We studied comorbidities in 6,480 HF patients enrolled (1 October 2012 and 6 October 2016) in the Asian Sudden Cardiac Death in Heart Failure (ASIAN-HF) registry. The prospective study design of the ASIAN-HF registry has been published previously [4,14]. The primary analysis described in the prospective study design related to sudden cardiac death and utilization of implantable cardiac defibrillators in HFrEF, and the primary outcomes have been published [15]. Subsequent publications from the ASIAN-HF registry are guided by a publication charter and overseen by a publications committee.

In brief, the ASIAN-HF registry is a multinational registry of Asian patients with HF from 46 medical centers across 11 regions (Taiwan, Hong Kong, China, India, Malaysia, Thailand, Singapore, Indonesia, Philippines, Japan, and Korea; S1 Table). Patients included in the ASIAN-HF registry were all eligible patients at enrollment sites who met predetermined inclusion and exclusion criteria and provided informed written consent for participation. Recruitment sites were selected to include a broad spectrum of medical, cardiology, and HF specialty units that regularly manage and follow patients with chronic HF. Patients included in the ASIAN-HF registry were >18 years of age with symptomatic HF (at least 1 previous episode of decompensated HF in the previous 6 months resulting in a hospital admission or treatment in outpatient clinic). Patients with severe valvular heart disease as a cause of HF, with a life-threatening comorbidity with a life expectancy <1 year, or unable or unwilling to give consent were excluded. The ASIAN-HF registry was originally designed to include only patients with HFrEF (LVEF < 40%) [4,14], but in 2013 the study underwent a protocol amendment to also include patients with HFpEF (LVEF ≥ 50%). Recruitment of patients with HFpEF started later than the recruitment of patients with HFrEF, for funding reasons. However, the delay was only 1 year (1 October 2012 versus 9 September 2013), and for the majority of the recruitment period (until 6 October 2016) there was overlap in recruitment of both types of HF. We do not anticipate that there were substantial shifts in epidemiology or treatment of patients with HFrEF or HFpEF during this short period of 1 year that may have biased the regional patterns of multimorbidity groups, although the potential for bias cannot be excluded. Data on demographics, previous medical history, clinical symptoms, and functional status were collected. According to the protocol, patients underwent standard 12-lead electrocardiography (ECG) and transthoracic echocardiography at inclusion.

### Study definitions

The definitions of comorbidities in the ASIAN-HF registry have previously been described [4,14]. Obesity was defined according to the standard body mass index (BMI) cutoff defined by the World Health Organization (WHO) (≥30 kg/m^2^). Coronary artery disease (CAD) was defined as angiographically documented presence of significant coronary obstruction, history of myocardial infarction, or prior revascularization. Hypertension was defined as any past or current history of hypertension and treatment for hypertension. Diabetes was defined as having a (prior) diagnosis of diabetes. Estimated glomerular filtration rate (eGFR) was calculated using the Modification of Diet in Renal Disease Study equation, and chronic kidney disease (CKD) was defined as eGFR < 60 ml/min/1.73 m^2^. Anemia was defined according to WHO criteria: hemoglobin <13 g/dl for men and <12 g/dl for women. Atrial fibrillation (AF) was defined as having a medical history or AF on ECG. Peripheral arterial and venous disease (PAVD), previous stroke, chronic obstructive pulmonary disease (COPD), peptic ulcer, renal artery stenosis, dementia, liver disease, cancer, and depression were identified by medical history.

QoL was measured using the Kansas City Cardiomyopathy Questionnaire (KCCQ), a 23-item self-administered HF-specific questionnaire validated in multiple HF-related disease states [16–21]. KCCQ domain scores range from 0 to 100; higher scores represent better QoL. Ethnicity was self-reported. Region income level was defined according to World Bank criteria: low—Indonesia, Philippines, and India; middle—China, Thailand, and Malaysia; high—Singapore, Hong Kong, Taiwan, South Korea, and Japan.

Medications by therapeutic class were identified, including angiotensin-converting enzyme inhibitors (ACEis) or angiotensin receptor blockers (ARBs), beta-blockers, mineralocorticoid receptor antagonists (MRAs), and diuretics. Medication use was captured at baseline.

### Outcomes

The primary outcome of this study was all-cause death or HF hospitalization within 1 year. In all, 5,875 (90.7%) patients had outcome data available, whereas 605 (9.3%) patients were lost to follow-up. Patients with less than 1 year of follow-up available were censored at their last known visit date. Outcomes were adjudicated by an independent committee. Secondary outcomes were all-cause mortality alone and hospitalization for HF alone. All data were captured prospectively in an electronic database, with registry operations and data management handled by Quintiles Outcomes as the contract research organization appointed by the academic executive committee.

### Echocardiography

The collection and processing of echocardiographic data has previously been described [14]. Echocardiography was performed at each center according to internationally accepted guidelines [22]. LVEF, LV dimensions, left atrial dimensions, LV diastolic function, stroke volume, and cardiac output were measured. The Cardiovascular Imaging Core Laboratory of the National University Health System, Singapore, provided oversight and imaging protocol guidelines as well as quality assurance of echocardiograms. Accuracy and reproducibility of interpreted results were ensured through consistent training and systematic analytical processes provided by the core laboratory according to international guidelines [22]. For further calculations, LV mass (LVM) was calculated from linear dimensions and indexed to height^2.7^ as well as to body surface area (BSA) [22]. Relative wall thickness (RWT) was calculated by the formula (2 × diastolic posterior wall thickness)/diastolic LV internal diameter. LV hypertrophy (LVH) was determined as LVM indexed to BSA >115 g/m^2^ in men and >95 g/m^2^ in women [22]. Normal cardiac geometry was defined as having no LVH and a RWT ≤ 0.42. Abnormal cardiac geometry (cardiac remodeling) was classified as concentric remodeling (no LVH and RWT > 0.42), concentric hypertrophy (LVH and RWT > 0.42), or eccentric hypertrophy (LVH and RWT ≤ 0.42). Left atrial size was indexed to BSA [22].

### Statistical analysis

Latent class analysis (LCA) was performed using the poLCA package in the R statistical package [23] to identify groups of patients with patterns of comorbidities. Briefly, a comprehensive list of comorbidities—which included AF, CAD, stroke, CKD, obesity, hypertension, COPD, peptic ulcer, renal artery stenosis, cancer, liver disease, dementia, anemia, depression, diabetes, and PAVD—was analyzed to identify group membership of individual patients. Maximum likelihood estimations were used to identify patient groups based on multimorbidity type for a range of 2–10 groups. The optimal number of groups was identified using the first minimum of the Bayesian information criterion (BIC). The BIC is suggested to provide for the most parsimonious model selection and is recommended in LCA [23–25]. poLCA uses random starts; therefore, each model was estimated with 10 replications. Cases with missing covariates were removed in this process. In this study, the optimal number of classes was 5 (S2 Table). Patients’ individual class membership was then derived using a Bayesian approach [23]. After determining the optimal number of clusters, the partial probabilities were averaged over the 10 replications. These partial probabilities were then used to calculate each group membership in a Bayesian fashion using the probabilities listed in S3 Table. By multiplying each probability corresponding to each variable, a patient’s probability of belonging to a group was determined. Final group selection was based on the patient’s highest probability of a group. Baseline echocardiographic characteristics and KCCQ domain scores were stratified according to group membership and are presented as means and standard deviations, medians and IQRs, or numbers and percentages, as appropriate. Differences between multimorbidity groups in the entire HF cohort were tested with 1-way analysis of variance (ANOVA), Kruskal–Wallis test, or the χ^2^ test, where appropriate. We corrected for multiple testing in the tables using the Benjamini–Hochberg correction, using a false discovery rate of 0.05. In addition, we tested for interaction between group membership and HF type (HFrEF or HFpEF) and stratified our analyses by HF type in the presence of significant interaction. For logistic regressions, the young group was used as the referent. In logistic regression, we further corrected for age, sex, inpatient versus outpatient enrollment, ethnicity, and New York Heart Association (NYHA) class. Kaplan–Meier curves stratified by group membership are shown, with differences between groups tested using the log-rank test for survival. Multivariable Cox regression analysis was used to test for differences between multimorbidity groups in all-cause mortality and HF-related hospitalization within 1 year, with the young group used as a referent. We corrected for confounders selected based on clinical considerations in a stepwise manner. In model 1 we corrected for age and sex. In model 2 we corrected for variables included in model 1 and geographic zone, previous hospitalization for HF (yes/no), NYHA class, and HFrEF versus HFpEF. In model 3 we corrected for all variables in model 2 and usage of ACEis/ARBs, beta-blockers, MRAs, and diuretics at baseline. When analyzing HF hospitalizations alone, all-cause mortality was used as a competing risk.

Prior to performing this study, we planned LCA and analyses regarding the differences between possible multimorbidity groups for the primary combined outcome as well as differences in clinical characteristics and echocardiographic parameters and regional distribution of multimorbidity groups. Based on recommendations during the peer-review process, we conducted additional sensitivity analyses investigating the differences between multimorbidity groups within a single ethnicity (Chinese) between 2 zones: Northeast Asia (South Korea, Japan, Taiwan, Hong Kong, and China) and Southeast Asia (Thailand, Malaysia, Philippines, Indonesia, and Singapore). Additionally, we included analyses of all-cause mortality alone and hospitalizations for HF alone (with all-cause mortality as a competing risk) based on recommendations from the peer-review process.

All tests were performed 2-sided, and *p*-values of <0.05 were considered statistically significant. Statistical analyses were performed using STATA 13.0 (StataCorp, College Station, TX, US) and R version 3.4.

## Results

### Multimorbidity groups identified by LCA

Overall, patients were on average 62 years old, and 27% were female (Table 1). Patients were primarily of Chinese (33%) and Indian (30%) ethnicity, and the majority of patients were in NYHA class II or III. The median number of comorbidities was 3, and 81% of patients had ≥2 comorbidities in addition to HF. Among all comorbidities, hypertension (55%) was the most common, followed by CAD (46%) and CKD (45%).

**Table 1:** Baseline characteristics according to multimorbidity group.

Characteristic	Total cohort	Multimorbidity group					*p*-Value
		Elderly/AF	Metabolic	Young	Ischemic	Lean diabetic	
***N***	6,480	1,048	1,129	1,759	1,261	1,283	** **
**Demographics**	** **						** **
Age, years	61.6 (13.3)	68.2 (12.4)	59.1 (12.5)	55.6 (14.3)	62.4 (11.3)	66.1 (10.8)	<0.001*
Female sex	1,750 (27.0%)	321 (30.6%)	302 (26.7%)	527 (30.0%)	217 (17.2%)	383 (29.9%)	<0.001*
Ethnicity							
Chinese	2,150 (33.2%)	437 (41.7%)	423 (37.5%)	484 (27.5%)	331 (26.3%)	475 (37.0%)	<0.001*
Indian	1,963 (30.3%)	87 (8.3%)	317 (28.1%)	670 (38.1%)	564 (44.8%)	325 (25.3%)	
Malay	973 (15.0%)	128 (12.2%)	200 (17.7%)	168 (9.6%)	190 (15.1%)	287 (22.4%)	
Japanese	661 (10.2%)	220 (21.0%)	82 (7.3%)	212 (12.1%)	68 (5.4%)	79 (6.2%)	
Korean	355 (5.5%)	109 (10.4%)	45 (4.0%)	121 (6.9%)	42 (3.3%)	38 (3.0%)	
Thai	199 (3.1%)	41 (3.9%)	26 (2.3%)	55 (3.1%)	33 (2.6%)	44 (3.4%)	
Filipino	53 (0.8%)	9 (0.9%)	13 (1.2%)	13 (0.7%)	8 (0.6%)	10 (0.8%)	
Indigenous SEA	109 (1.7%)	17 (1.6%)	19 (1.7%)	31 (1.8%)	22 (1.7%)	20 (1.6%)	
Other	14 (0.2%)	0 (0.0%)	3 (0.3%)	5 (0.3%)	1 (0.1%)	5 (0.4%)	
County income level							
Low	2,089 (32.3%)	141 (13.5%)	311 (27.5%)	753 (42.8%)	606 (48.1%)	278 (21.7%)	<0.001*
Middle	1,289 (19.9%)	198 (18.9%)	236 (20.9%)	375 (21.3%)	249 (19.7%)	231 (18.0%)	
High	3,102 (47.8%)	709 (67.7%)	582 (51.6%)	631 (35.9%)	406 (32.2%)	774 (60.3%)	
NYHA class							
I	776 (13.5%)	115 (12.2%)	145 (14.6%)	214 (13.7%)	153 (13.3%)	149 (13.3%)	0.014*
II	3,085 (53.5%)	512 (54.4%)	544 (54.8%)	855 (54.8%)	590 (51.2%)	584 (52.2%)	
III	1,586 (27.5%)	247 (26.2%)	269 (27.1%)	400 (25.7%)	336 (29.2%)	334 (29.8%)	
IV	317 (5.5%)	67 (7.1%)	35 (3.5%)	90 (5.8%)	73 (6.3%)	52 (4.6%)	
Systolic blood pressure, mm Hg	121.0 (21.2)	119.7 (21.2)	126.7 (21.1)	116.7 (19.5)	116.8 (20.2)	126.9 (21.7)	<0.001*
Diastolic blood pressure, mm Hg	72.4 (12.6)	71.2 (13.3)	76.2 (13.4)	72.6 (12.4)	70.9 (11.7)	71.2 (12.1)	<0.001*
Body mass index, kg/m^2^	25.3 (5.3)	23.4 (3.7)	29.0 (6.6)	24.6 (5.2)	23.6 (3.2)	26.2 (5.3)	<0.001*
Heart rate, bpm	79.4 (16.3)	78.3 (17.0)	80.8 (16.7)	80.0 (17.3)	78.9 (14.6)	78.9 (15.6)	0.005*
eGFR, ml/min/1.73 m^2^	65.0 (28.1)	57.1 (22.8)	77.7 (22.8)	80.5 (26.8)	64.4 (27.4)	43.1 (20.4)	<0.001*
Ischemic etiology	2,840 (47.2%)	339 (34.9%)	450 (43.1%)	353 (22.2%)	853 (70.8%)	845 (69.8%)	<0.001*
Previous HF hospitalization							
No	2,182 (33.9%)	331 (31.6%)	445 (39.5%)	666 (38.5%)	414 (32.9%)	326 (25.5%)	<0.001*
Yes	3,777 (58.6%)	655 (62.6%)	624 (55.4%)	855 (49.4%)	766 (60.8%)	877 (68.7%)	
Unknown	483 (7.5%)	61 (5.8%)	58 (5.1%)	211 (12.2%)	80 (6.3%)	73 (5.7%)	
**Signs and symptoms of HF**	** **						
Shortness of breath at exertion	4,647 (72.0%)	733 (69.9%)	771 (68.4%)	1,268 (72.9%)	952 (75.5%)	923 (72.2%)	0.001*
Shortness of breath at rest	1,107 (17.2%)	178 (17.0%)	202 (17.9%)	278 (16.0%)	206 (16.3%)	243 (19.0%)	0.21
Reduction in exercise tolerance	4,384 (67.9%)	692 (66.0%)	746 (66.1%)	1,186 (68.2%)	900 (71.4%)	860 (67.3%)	0.033*
Nocturnal cough	1,118 (17.3%)	172 (16.4%)	175 (15.5%)	303 (17.4%)	219 (17.4%)	249 (19.5%)	0.12
Orthopnea	1,368 (21.2%)	223 (21.3%)	237 (21.0%)	316 (18.2%)	258 (20.5%)	334 (26.1%)	<0.001*
Paroxysmal nocturnal dyspnea	1,121 (17.4%)	172 (16.4%)	188 (16.7%)	289 (16.6%)	216 (17.1%)	256 (20.0%)	0.089
Angina	697 (10.8%)	95 (9.1%)	131 (11.6%)	136 (7.8%)	177 (14.0%)	158 (12.4%)	<0.001*
Elevated JVP	940 (14.6%)	166 (15.8%)	152 (13.5%)	173 (10.0%)	185 (14.7%)	264 (20.7%)	<0.001*
Peripheral edema	1,627 (25.2%)	287 (27.4%)	330 (29.3%)	294 (16.9%)	270 (21.4%)	446 (35.0%)	<0.001*
Plural rales	1,062 (16.5%)	168 (16.0%)	180 (16.0%)	188 (10.8%)	241 (19.1%)	285 (22.3%)	<0.001*
**Medical history**	** **						
Previous VT/VF	445 (6.9%)	111 (10.6%)	54 (4.8%)	126 (7.3%)	79 (6.3%)	75 (5.9%)	<0.001*
Obesity	912 (15.3%)	19 (2.0%)	448 (45.1%)	189 (11.6%)	0 (0.0%)	256 (21.6%)	<0.001*
CAD	2,975 (46.2%)	306 (29.2%)	405 (36.0%)	193 (11.2%)	1,102 (87.5%)	969 (75.9%)	<0.001*
Diabetes	2,656 (41.3%)	164 (15.6%)	716 (63.5%)	1 (0.1%)	540 (42.9%)	1,235 (96.7%)	<0.001*
CKD	2,312 (45.4%)	585 (63.6%)	137 (14.7%)	200 (16.1%)	442 (47.7%)	948 (89.1%)	<0.001*
Stroke	434 (6.7%)	207 (19.8%)	59 (5.2%)	0 (0.0%)	2 (0.2%)	166 (13.0%)	<0.001*
Atrial fibrillation	1,279 (19.9%)	708 (67.6%)	124 (11.0%)	200 (11.6%)	0 (0.0%)	247 (19.3%)	<0.001*
Hypertension	3,562 (55.4%)	683 (65.2%)	990 (87.8%)	249 (14.4%)	430 (34.1%)	1,210 (94.8%)	<0.001*
PAVD	203 (3.2%)	37 (3.5%)	9 (0.8%)	7 (0.4%)	30 (2.4%)	120 (9.4%)	<0.001*
COPD	542 (8.4%)	145 (13.8%)	105 (9.3%)	92 (5.3%)	86 (6.8%)	114 (8.9%)	<0.001*
Peptic ulcer	213 (3.3%)	87 (8.3%)	32 (2.8%)	3 (0.2%)	15 (1.2%)	76 (6.0%)	<0.001*
Renal artery stenosis	55 (0.9%)	19 (1.8%)	0 (0.0%)	0 (0.0%)	15 (1.2%)	21 (1.6%)	<0.001*
Liver disease	199 (3.1%)	87 (8.3%)	4 (0.4%)	42 (2.4%)	14 (1.1%)	52 (4.1%)	<0.001*
Cancer	216 (3.4%)	100 (9.5%)	10 (0.9%)	43 (2.5%)	2 (0.2%)	61 (4.8%)	<0.001*
Dementia	43 (0.7%)	24 (2.3%)	1 (0.1%)	0 (0.0%)	2 (0.2%)	16 (1.3%)	<0.001*
Anemia	2,066 (40.6%)	350 (40.6%)	95 (11.6%)	259 (22.0%)	575 (69.2%)	787 (78.5%)	<0.001*
Depression	79 (1.2%)	34 (3.2%)	6 (0.5%)	4 (0.2%)	0 (0.0%)	35 (2.7%)	<0.001*
Smoker	2,636 (41.0%)	464 (44.3%)	488 (43.3%)	600 (34.8%)	549 (43.6%)	535 (41.9%)	<0.001*
Alcohol	1,697 (26.4%)	322 (30.8%)	341 (30.3%)	456 (26.4%)	292 (23.2%)	286 (22.4%)	<0.001*
**Number of comorbidities**	3 (2, 4)	3 (3, 4)	3 (2, 4)	1 (0, 1)	3 (2, 4)	5 (5, 6)	<0.001*
**Medications**	** **						
ACEi or ARB	4,562 (73.7%)	713 (70.1%)	904 (83.2%)	1,265 (78.3%)	881 (72.1%)	799 (64.1%)	<0.001*
Beta-blocker	4,682 (75.7%)	774 (76.1%)	840 (77.3%)	1,224 (75.8%)	894 (73.2%)	950 (76.2%)	0.20
MRA	3,222 (52.1%)	502 (49.4%)	574 (52.8%)	1,015 (62.8%)	654 (53.5%)	477 (38.3%)	<0.001*
Diuretic	4,960 (80.2%)	831 (81.7%)	873 (80.3%)	1,251 (77.5%)	959 (78.5%)	1,046 (83.9%)	<0.001*
**Laboratory tests**	** **						
Potassium, mmol/l	4.2 (3.9, 4.6)	4.2 (3.9, 4.6)	4.1 (3.8, 4.5)	4.2 (3.9, 4.5)	4.2 (3.9, 4.6)	4.3 (3.9, 4.6)	0.002*
Sodium, mmol/l	139 (136, 141)	139 (136, 141)	139 (136, 141)	139 (137, 141)	138 (135, 141)	138 (136, 140)	<0.001*
Creatinine, μmol/l	97.3 (79.6, 131.0)	106.1 (88.4, 137.0)	84.0 (70.7, 99.0)	82.0 (70.7, 97.3)	100.0 (79.6, 132.6)	138.0 (112.0, 185.0)	<0.001*

Data are given as mean (SD), median (IQR), or number (percent).

*Significant after Benjamini–Hochberg correction using a false discovery rate of 0.05.

ACEi, angiotensin-converting enzyme inhibitor; ARB, angiotensin receptor blocker; bpm, beats per minute; CAD, coronary artery disease; CKD, chronic kidney disease; COPD, chronic obstructive pulmonary disease; eGFR, estimated glomerular filtration rate; HF, heart failure; JVP, jugular venous pressure; MRA, mineralocorticoid receptor antagonist; PAVD, peripheral arterial and venous disease; NYHA, New York Heart Association; SEA, Southeast Asian; VF, ventricular fibrillation; VT, ventricular tachycardia.

In the entire cohort, 5 multimorbidity groups of relatively equal size (*N* = 1,048–1,759) were identified, each characterized by a different combination of comorbidities: elderly/AF, metabolic, young, ischemic, and lean diabetic.

Patients in the elderly/AF group, were the oldest (mean age 68.2 years), had the highest prevalence of AF (67.6%) and stroke (19.8%), and had a comparatively high prevalence of CKD (63.6%). They were also more likely to be of Chinese, Japanese, or Korean ethnicity and from high-income regions.

Patients in the metabolic group had the highest mean BMI (29 kg/m^2^) and prevalence of obesity (45.1%), combined with a high prevalence of hypertension (87.8%) and diabetes (63.5%). These patients were relatively young (mean age 59.1 years), often of Malay ethnicity, and most often on ACEis/ARBs.

Patients in the young group were the youngest (mean age 55.6 years) and had an exceptionally low proportion of all comorbidities, with high prevalence on non-ischemic etiology of HF (77.8%). These patients were primarily of Indian or Chinese ethnicity and from low-income regions and were most the likely to be treated with MRAs. These patients had the lowest absolute number of comorbidities (0, IQR 0,1).

Patients in the ischemic group were of intermediate age (mean age 62.4 years); this group had the highest proportion of men (83%) compared to the other groups. Overall, these patients were most often Indian and had ischemic etiology of HF (71%), with lower prevalence of diabetes (43%) compared to the young group but the highest prevalence of CAD (88%; *p* for all comparisons < 0.001) and high prevalence of anemia (69%).

The lean diabetic group consisted of patients of intermediate age (mean age 66.1 years) with a strikingly high prevalence of diabetes (97%) despite a low prevalence of obesity (22%). They also had high prevalence of hypertension (95%), CKD (89%), anemia (78.5%), and CAD (76%). These patients were commonly of Malay ethnicity and from high-income regions (60%). These also appeared to be the sickest patients, with the worst signs and symptoms of HF and frequent history of hospitalization for HF (69%). These patients had the highest absolute number of comorbidities (5, IQR 5, 6).

Patients from the lean diabetic group had the worst overall QoL, while patients from the young group had the best QoL, comparing overall summary scores (Table 2). Similarly, the lean diabetic group had poorer QoL as compared to the young group in the domains of total symptoms and social limitations.

**Table 2:** Kansas City Cardiomyopathy Questionnaire domain scores according to multimorbidity group.

Domain	Elderly/AF, *N* = 1,048	Metabolic, *N* = 1,129	Young, *N* = 1,759	Ischemic, *N* = 1,261	Lean diabetic, *N* = 1,283	*p*-Value
Physical limitation	71 (50, 92)	79 (58, 92)	75 (55, 92)	71 (50, 88)	67 (46, 88)	<0.001*
Symptom stability	50 (50, 75)	50 (50, 75)	50 (50, 75)	50 (50, 75)	50 (50, 75)	<0.001*
Symptom frequency	75 (52, 92)	75 (50, 94)	75 (56, 94)	75 (52, 92)	69 (40, 88)	<0.001*
Symptom burden	75 (58, 100)	83 (58, 100)	83 (58, 100)	75 (58, 92)	75 (50, 92)	<0.001*
Total symptom score	76 (53, 94)	77 (54, 94)	77 (58, 94)	75 (55, 92)	71 (46, 90)	<0.001*
Self-efficacy score	63 (50, 75)	75 (50, 88)	75 (50, 88)	75 (50, 88)	75 (50, 88)	<0.001*
Quality of life score	58 (42, 75)	58 (42, 83)	58 (42, 83)	58 (42, 75)	58 (33, 75)	<0.001*
Social limitation score	67 (31, 94)	75 (50, 100)	75 (42, 100)	69 (38, 92)	58 (25, 88)	<0.001*
Overall summary score	67 (49, 85)	71 (52, 86)	72 (51, 86)	69 (48, 83)	62 (42, 81)	<0.001*
Clinical summary score	72 (53, 90)	76 (57, 92)	76 (57, 91)	72 (54, 88)	68 (46, 85)	<0.001*

*Significant after Benjamini–Hochberg correction using a false discovery rate of 0.05.

AF, atrial fibrillation.

### Distribution of multimorbidity groups by region

**Fig 1:**
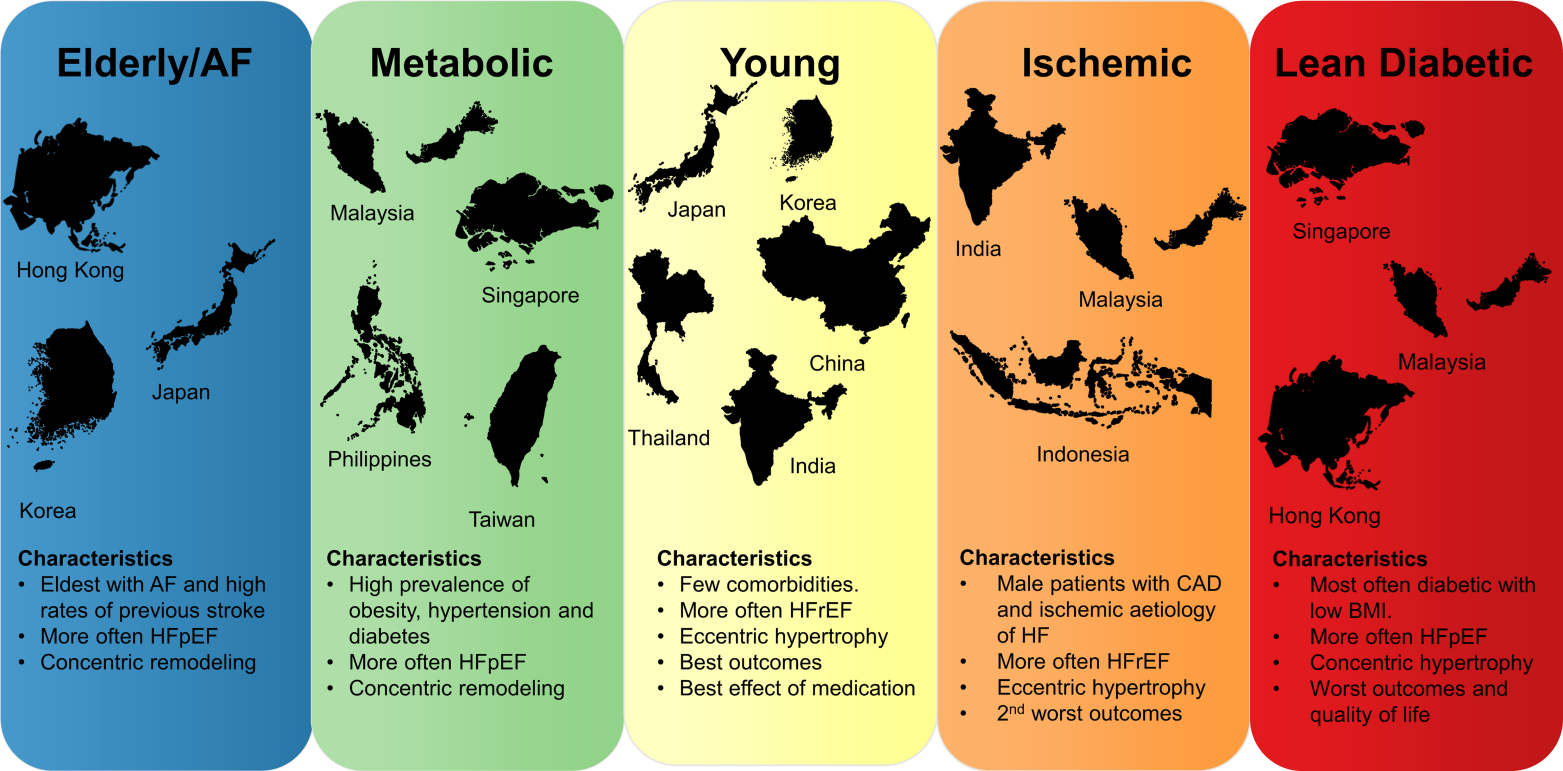
Concept figure summarizing the most important findings of this study. Region sizes in the figure are not to scale. AF, atrial fibrillation; BMI, body mass index; CAD, coronary artery disease; HF, heart failure; HFpEF, heart failure with preserved ejection fraction; HFrEF, heart failure with reduced ejection fraction.

**Fig 2:**
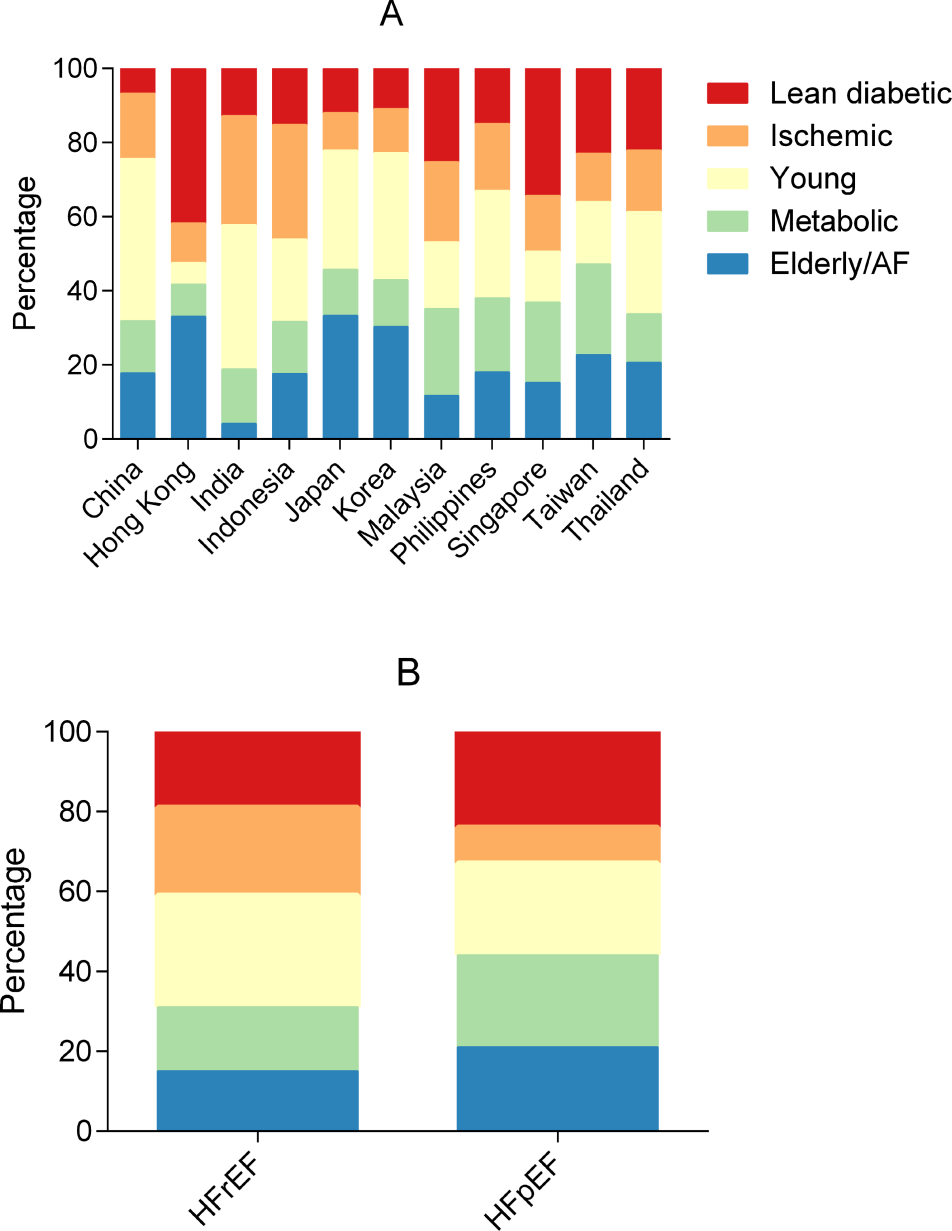
Bar graphs showing the distribution of multimorbidity groups across regions and HFrEF/HFpEF. By region (A) and HFrEF versus HFpEF (B). AF, atrial fibrillation; HFpEF, heart failure with preserved ejection fraction; HFrEF, heart failure with reduced ejection fraction.

The distribution of multimorbidity groups by region is summarized in Fig 1. In China and Thailand, the young group was most prevalent (Fig 2A). In Hong Kong, the majority of patients belonged to the lean diabetic and elderly/AF groups. Indian and Indonesian patients most often belonged to the young and ischemic groups. Japanese and Korean patients most often belonged to the elderly/AF and young groups. Overall, patients from Singapore and Malaysia were in either the lean diabetic or metabolic group. Patients from the Philippines and Taiwan were most often in the metabolic group.

We performed sensitivity testing restricted to a single ethnicity (Chinese) in 2 different zones (Southeast Asia and Northeast Asia) and found that the same phenotypic groups emerged in each zone, with similar characteristics within each group. This suggests that the phenotypic groups were not simply due to ethnic or regional differences in inclusion criteria, but may represent underlying biological differences.

### Distribution of multimorbidity groups by type of HF

The relative prevalence of the ischemic and young groups was higher in HFrEF, while the elderly/AF, metabolic, and lean diabetic groups had a higher relative prevalence in HFpEF (Fig 2B). When adjusted for age, sex, inpatient versus outpatient enrollment, ethnicity, and NYHA class, patients in the metabolic group were more likely to have HFpEF, while patients in the ischemic group were more likely to have HFrEF.

### Differences in cardiac structure and function by multimorbidity group

**Fig 3:**
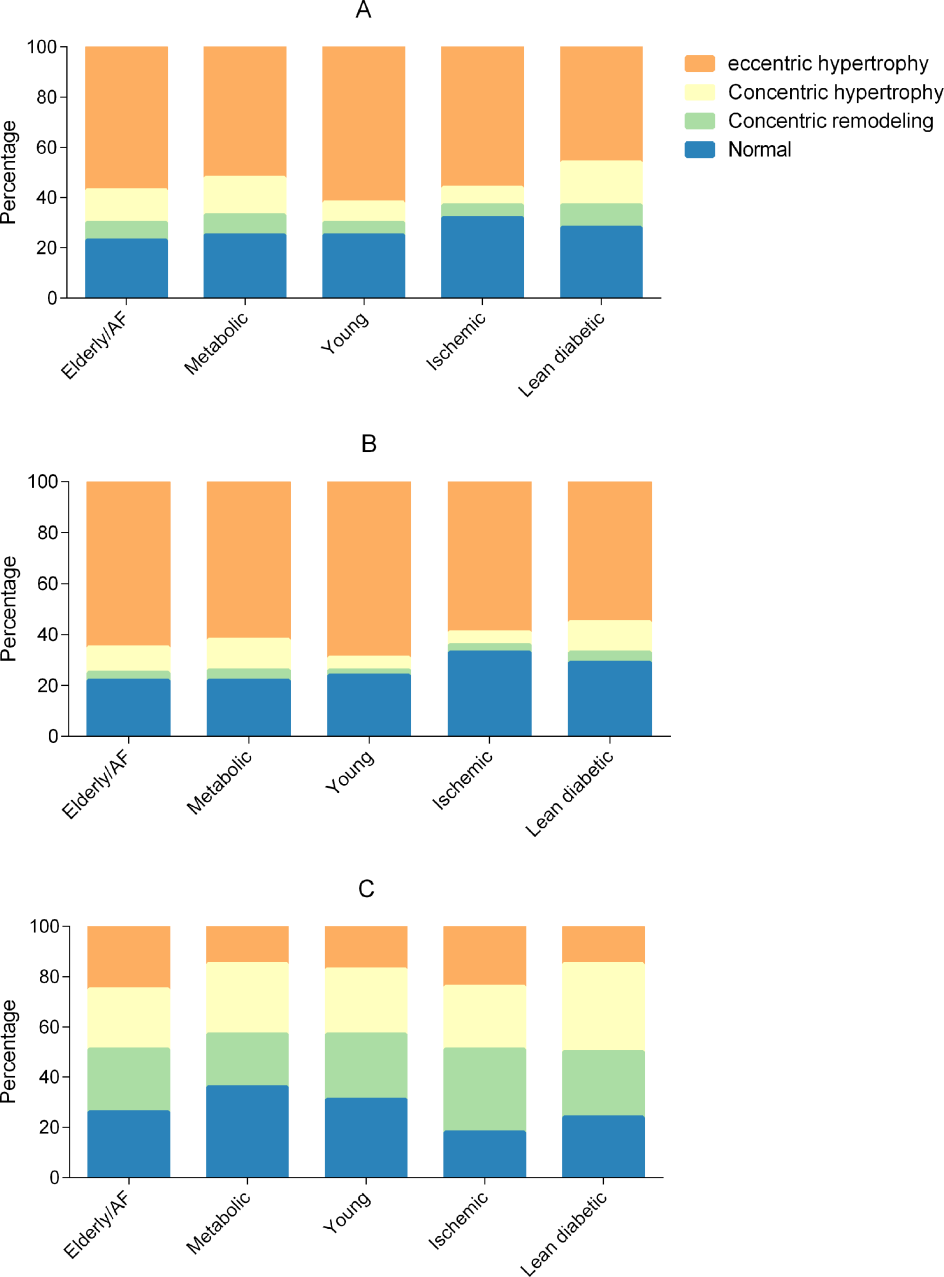
Bar graphs showing cardiac geometry across multimorbidity groups. Total cohort (A), heart failure with reduced ejection fraction (B), and heart failure with preserved ejection fraction (C). AF, atrial fibrillation.

Overall, the metabolic and lean diabetic groups had the highest proportions of concentric hypertrophy, and the young group had the highest proportion of eccentric hypertrophy (Fig 3A). When correcting for age, sex, inpatient versus outpatient enrollment, ethnicity, NYHA class, and HFrEF versus HFpEF, the ischemic group (odds ratio [OR] 0.73, 95% CI 0.61–0.87) and lean diabetic group (OR 0.71, 95% CI 0.59–0.85) had less LVH compared to the young group. In contrast to the young group, the elderly/AF (OR 1.91, 95% CI 1.52–2.40), metabolic (OR 2.46, 95% CI 1.98–3.06), and lean diabetic (OR 2.59, 95% CI 2.09–3.21) groups had more concentric remodeling after multivariable correction.

Echocardiographic data stratified by multimorbidity group and HF type (HFrEF or HFpEF) are presented in Tables 3 and 4. To study whether multimorbidity group affected cardiac geometry differently in patients with HFrEF and HFpEF, we studied interactions between multimorbidity group and HF type (HFrEF or HFpEF). We observed a significant interaction between multimorbidity group and HF type for both concentric remodeling (*P* = 0.001) and LVH (*P* = 0.011). In HFrEF, the metabolic (OR 2.53, 95% CI 1.84–3.47) and lean diabetic (OR 2.39, 95% CI 1.72–3.33) groups were more likely to have concentric remodeling as compared to the young group, after adjusting for age, sex, inpatient versus outpatient enrollment, ethnicity, and NYHA class. The ischemic group was less likely to have LVH than the young group (OR 0.65, 95% CI 0.53–0.78). In HFrEF, the young group had the highest prevalence of eccentric hypertrophy, followed by the elderly/AF group. In HFpEF, the lean diabetic group had the highest proportion of concentric remodeling (Fig 3B and 3C).

**Table 3:** Echocardiographic characteristics.

Characteristic	Multimorbidity group					*p*-Value
	Elderly/AF, *N* = 1,048	Metabolic, *N* = 1,129	Young, *N* = 1,759	Ischemic, *N* = 1,261	Lean diabetic, *N* = 1,283	
**LV dimensions**						
LV end diastolic dimension, mm	58 (51, 66)	59 (52, 66)	61 (54, 68)	60 (55, 66)	57 (50, 63)	<0.001*
LV end systolic dimension, mm	48 (36, 56)	49 (36, 57)	52 (43, 60)	51 (43, 58)	46 (36, 54)	<0.001*
LV end diastolic volume, ml	139 (101, 192)	156 (111, 206)	174 (132, 223)	164 (128, 207)	137 (102, 179)	<0.001*
LV end systolic volume, ml	92 (52, 137)	104 (56, 151)	124 (84, 170)	115 (84, 154)	90 (55, 130)	<0.001*
LV end diastolic volume indexed to BSA, ml/m^2^	88 (64, 116)	86 (63, 111)	101 (79, 128)	97 (76, 122)	79 (61, 103)	<0.001*
LV end systolic volume indexed to BSA, ml/m^2^	60 (36, 83)	60 (39, 81)	72 (50, 97)	69 (50, 90)	53 (34, 74)	<0.001*
IVSD, mm	9.6 (8.0, 11.0)	10.0 (8.4, 12.0)	9.0 (8.0, 10.0)	9.0 (7.7, 10.0)	10.0 (8.2, 12.0)	<0.001*
PWTD, mm	9.5 (8.0, 11.0)	10.0 (9.0, 11.5)	9.0 (8.0, 10.0)	9.0 (8.0, 10.0)	10.0 (8.0, 11.0)	<0.001*
Relative wall thickness	0.32 (0.26, 0.40)	0.34 (0.28, 0.42)	0.30 (0.25, 0.36)	0.31 (0.26, 0.36)	0.34 (0.28, 0.43)	<0.001*
LV mass, g	213 (164, 276)	228 (180, 297)	225 (175, 283)	211 (174, 258)	210 (167, 264)	<0.001*
LV mass indexed to BSA, g/m^2^	132 (104, 165)	128 (100, 161)	130 (104, 164)	126 (102, 152)	122 (98, 152)	<0.001*
LV hypertrophy	555 (70.3%)	536 (67.4%)	919 (70.6%)	604 (63.7%)	565 (63.0%)	<0.001*
Concentric remodeling	183 (21.6%)	236 (26.1%)	173 (12.6%)	124 (12.4%)	263 (27.2%)	<0.001*
**Systolic function**						
LV ejection fraction, percent	31 (24, 39)	31 (23, 39)	29 (23, 36)	30 (23, 35)	32 (25, 39)	<0.001*
**Diastolic function**						
E wave, cm/s	81 (61, 103)	81 (62, 100)	75 (56, 96)	80 (61, 99)	88 (68, 108)	<0.001*
A wave, cm/s	62 (39, 83)	66 (40, 87)	59 (40, 77)	56 (35, 80)	69 (40, 87)	<0.001*
E′ medial, cm/s	4.8 (3.6, 6.0)	4.0 (3.3, 5.6)	4.6 (3.6, 6.0)	4.0 (3.0, 5.0)	4.0 (3.0, 5.0)	<0.001*
E/e′ ratio	17.1 (12.5, 23.6)	17.8 (13.4, 25.0)	15.5 (11.7, 21.2)	19.6 (14.3, 27.4)	20.3 (15.1, 27.3)	<0.001*
E/a′ ratio	1.1 (0.7, 2.2)	1.2 (0.8, 2.2)	1.2 (0.8, 2.1)	1.5 (0.8, 2.6)	1.3 (0.8, 2.4)	<0.001*
**LA dimensions**						
LA volume, ml	80 (57, 110)	63 (41, 87)	57 (37, 83)	57 (38, 81)	67 (48, 89)	<0.001*
LA volume indexed to BSA, ml/m^2^	50 (33, 66)	35 (23, 49)	34 (20, 49)	33 (23, 46)	39 (28, 51)	<0.001*

Data given as median (IQR) or number (percent).

*Significant after Benjamini–Hochberg correction using a false discovery rate of 0.05.

BSA, body surface area; IVSD, interventricular septal thickness in diastole; LA, left atrial; LV, left ventricle; PWTD, posterior wall thickness in diastole.

**Table 4:** Echocardiographic characteristics, stratified by multimorbidity groups and heart failure type.

Characteristic	Multimorbidity group					*p*-Value
	Elderly/AF	Metabolic	Young	Ischemic	Lean diabetic	
**HFrEF**						
*LV dimensions*						
LV end diastolic volume, ml	162 (126, 208)	176 (138, 228)	181 (142, 228)	171 (136, 211)	153 (121, 194)	<0.001*
LV end systolic volume, ml	113 (84, 154)	126 (95, 171)	130 (98, 175)	121 (94, 159)	106 (79, 142)	<0.001*
IVSD, mm	9.0 (8.0, 11.0)	9.7 (8.0, 11.0)	9.0 (8.0, 10.0)	9.0 (7.2, 10.0)	9.7 (8.0, 11.0)	<0.001*
PWTD, mm	9.0 (8.0, 11.0)	9.9 (8.6, 11.0)	9.0 (8.0, 10.0)	9.0 (8.0, 10.0)	9.5 (8.0, 11.0)	<0.001*
LV mass indexed to BSA, g/m^2^	140 (111, 171)	133 (109, 170)	133 (109, 166)	127 (105, 154)	128 (103, 157)	<0.001*
Relative wall thickness	0.3 (0.2, 0.4)	0.3 (0.3, 0.4)	0.3 (0.2, 0.3)	0.3 (0.3, 0.4)	0.3 (0.3, 0.4)	<0.001*
*Systolic function*						
LV ejection fraction, percent	28 (22, 34)	27 (21, 34)	27 (22, 32)	28 (22, 33)	28 (23, 34)	<0.001*
*Diastolic function*						
E wave, cm/s	80 (60, 103)	84 (64, 101)	75 (56, 96)	80 (62, 100)	88 (68, 106)	<0.001*
A wave, cm/s	59 (36, 79)	55 (34, 78)	58 (38, 76)	52 (34, 77)	59 (35, 82)	0.18
E′ medial, cm/s	4.5 (3.2, 5.6)	4.0 (3.0, 5.2)	4.4 (3.4, 6.0)	4.0 (3.0, 5.0)	4.0 (3.0, 5.0)	<0.001*
E/e′ ratio	17.9 (13.1, 24.3)	19.2 (14.2, 26.9)	15.8 (12.0, 21.7)	20.4 (14.5, 28.8)	21.8 (16.0, 29.8)	<0.001*
E/a′ ratio	1.2 (0.7, 2.3)	1.5 (0.8, 2.6)	1.3 (0.8, 2.2)	1.6 (0.8, 2.7)	1.6 (0.8, 2.8)	<0.001*
*Atrial size*						
LA volume indexed to BSA, ml/m^2^	49.6 (32.5, 66.8)	36.5 (24.7, 49.2)	34.3 (20.2, 49.0)	33.3 (22.9, 46.9)	40.6 (29.9, 52.2)	<0.001*
**HFpEF**						
*LV dimensions*	** **	** **	** **	** **	** **	
LV end diastolic volume, ml	88 (72, 113)	98 (77, 121)	98 (82, 137)	97 (79, 121)	89 (68, 120)	0.006*
LV end systolic volume, ml	35 (26, 49)	38 (29, 50)	41 (29, 60)	38 (30, 50)	36 (26, 55)	0.057
IVSD, mm	10.0 (9.0, 12.0)	10.5 (9.7, 12.0)	10.0 (9.0, 12.0)	10.0 (9.0, 12.0)	11.0 (9.9, 12.0)	0.005*
PWTD, mm	10.0 (9.0, 12.0)	10.3 (9.2, 12.0)	10.0 (9.0, 11.0)	10.0 (9.0, 11.0)	11.0 (9.0, 12.0)	0.001*
LV mass indexed to BSA, g/m^2^	103 (84, 129)	98 (83, 120)	99 (85, 130)	105 (85, 130)	105 (86, 134)	0.62
Relative wall thickness	0.4 (0.4, 0.5)	0.4 (0.4, 0.5)	0.4 (0.3, 0.5)	0.4 (0.4, 0.5)	0.4 (0.4, 0.5)	0.016*
*Systolic function*						
LV ejection fraction, percent	60 (55, 66)	60 (56, 68)	60 (55, 62)	60 (55, 64)	60 (55, 66)	<0.001*
*Diastolic function*						
E wave, cm/s	83 (63, 103)	73 (60, 95)	75 (59, 94)	78 (59, 92)	88 (68, 111)	<0.001*
A wave, cm/s	76 (52, 91)	84 (66, 99)	69 (54, 91)	75 (63, 93)	85 (70, 101)	<0.001*
E′ medial, cm/s	5.7 (4.1, 6.6)	5.0 (4.0, 6.9)	5.3 (4.3, 7.0)	5.0 (4.0, 6.3)	5.0 (4.0, 6.0)	0.084
E/e′ ratio	15.0 (11.6, 20.2)	15.0 (10.6, 19.9)	13.2 (10.0, 17.2)	14.9 (12.0, 19.4)	17.6 (14.2, 23.3)	<0.001*
E/a′ ratio	0.9 (0.7, 1.5)	0.8 (0.7, 1.1)	1.0 (0.8, 1.5)	1.0 (0.7, 1.3)	0.9 (0.7, 1.4)	0.036
*Atrial size*						
LA volume indexed to BSA, ml/m^2^	51.2 (34.6, 63.1)	29.9 (18.3, 41.4)	32.9 (24.0, 46.9)	30.8 (21.5, 40.2)	33.4 (25.2, 46.0)	<0.001*

Data given as median (IQR).

*Significant after Benjamini–Hochberg correction using a false discovery rate of 0.05.

BSA, body surface area; HFpEF, heart failure with preserved ejection fraction; HFrEF, heart failure with reduced ejection fraction; IVSD, interventricular septal thickness in diastole; LA, left atrial; LV, left ventricle; PWTD, posterior wall thickness in diastole.

### Outcomes by multimorbidity group

In the overall cohort, 1,125 (19.2%) patients experienced the primary combined outcome of all-cause mortality or hospitalization for HF within 1 year. Regarding secondary outcomes, 564 (9.6%) patients died, and 679 (11.6%) patients were hospitalized within 1 year.

**Fig 4:**
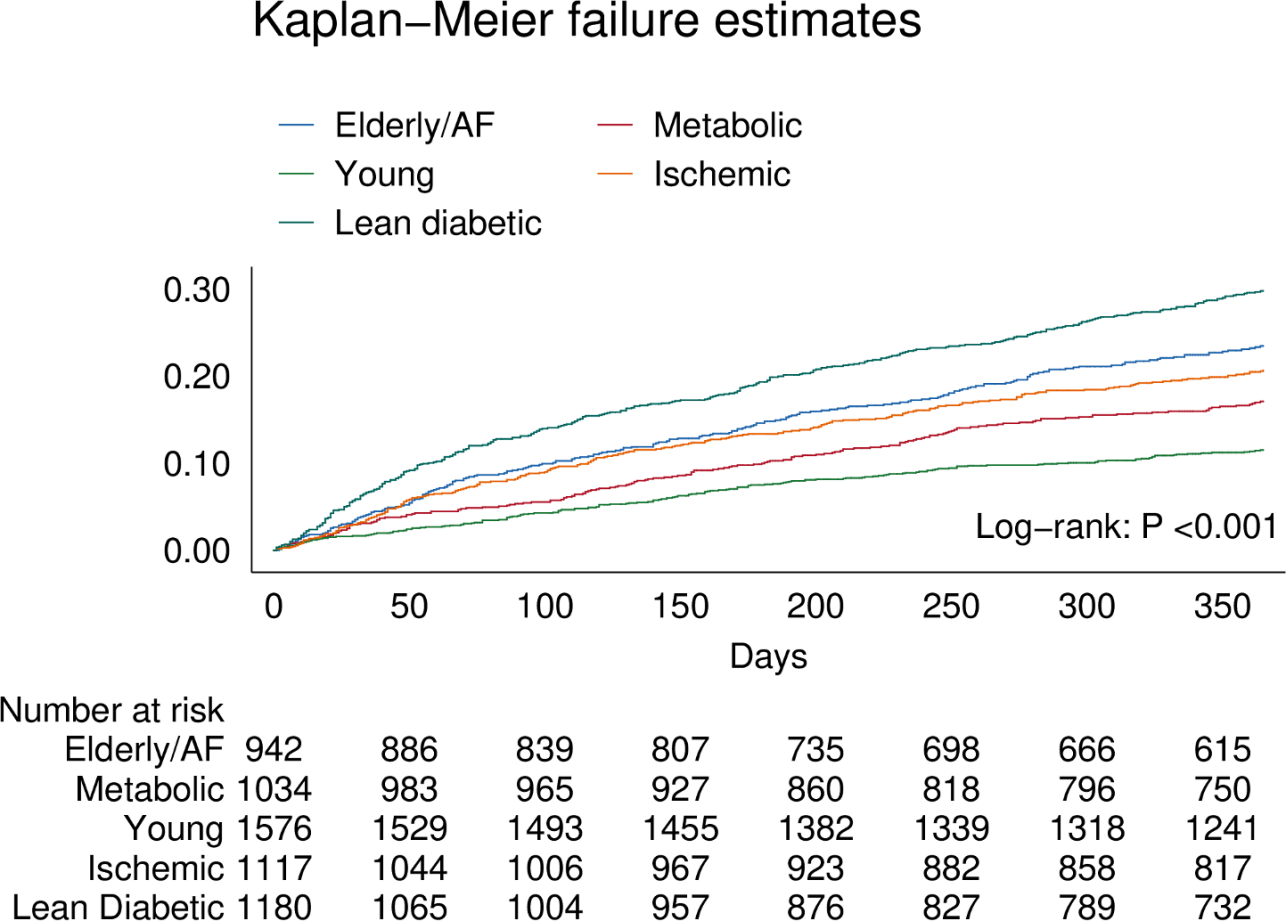
Kaplan–Meier curve showing differences for the primary combined outcome of all-cause mortality and HF-related hospitalization within 1 year across multimorbidity groups. AF, atrial fibrillation.

There were clear differences in the primary combined outcome between multimorbidity groups (*P* < 0.001; Fig 4). Particularly, the lean diabetic group had the highest proportion of events of the combined outcome (HF hospitalization or mortality) within 1 year (29%), while the young group had the lowest (11%). In model 3, the lean diabetic group remained associated with the highest proportion of events of the combined outcome (hazard ratio [HR] 1.79, 95% CI 1.46–2.22) compared to the young group (Table 5). Similarly, the elderly/AF (HR 1.57, 95% CI 1.26–1.96), metabolic (HR 1.28, 95% CI 1.02–1.60), and ischemic groups (HR 1.52, 95% CI 1.22–1.88) had higher rates of the combined outcome than the young group. Differences in survival remained after adjusting for systolic function (LVEF), diastolic function (E/e′), and cardiac geometry across groups. After correcting for number of comorbidities, the predictive power of multimorbidity group remained; here particularly the ischemic group was associated with a higher proportion of the combined outcome (HR 1.47, 95% CI 1.08–1.99). When investigating mortality alone, the elderly/AF group had the highest hazards for dying within 1 year (HR 1.71, 95% CI 1.26–2.32). For hospitalizations for HF, the lean diabetic group had the highest hazards (HR 1.99, 95% CI 1.52–2.60).

**Table 5:** Results of Cox regression analysis across multimorbidity groups for the combined outcome of all-cause mortality and hospitalization for heart failure, all-cause mortality alone, and hospitalization for heart failure alone.

Outcome and multimorbidity group	Cases/*N*	Hazard ratio (95% CI)			
		Univariable	Model 1	Model 2	Model 3
**Combined outcome**					
Young	177/1,580	Ref	Ref	Ref	Ref
Elderly/AF	211/943	2.17 (1.78–2.65)	2.11 (1.71–2.60)	1.58 (1.27–1.96)	1.57 (1.26–1.96)
Metabolic	170/1,037	1.52 (1.23–1.87)	1.50 (1.21–1.85)	1.26 (1.01–1.57)	1.28 (1.02–1.60)
Ischemic	229/1,122	1.89 (1.56–2.31)	1.80 (1.48–2.21)	1.53 (1.24–1.89)	1.51 (1.22–1.88)
Lean diabetic	338/1,185	2.89 (2.41–3.47)	2.83 (2.34–3.41)	1.89 (1.54–2.32)	1.79 (1.46–2.22)
**All-cause mortality**					
Young	96/1,580	Ref	Ref	Ref	Ref
Elderly/AF	115/943	2.11 (1.61–2.77)	1.98 (1.49–2.63)	1.68 (1.24–2.26)	1.71 (1.26–2.32)
Metabolic	66/1,037	1.05 (0.77–1.44)	1.02 (0.74–1.40)	0.88 (0.63–1.22)	0.88 (0.62–1.24)
Ischemic	127/1,122	1.89 (1.45–2.46)	1.77 (1.35–2.33)	1.35 (1.01–1.80)	1.34 (1.01–1.80)
Lean diabetic	159/1,185	2.34 (1.81–3.01)	2.21 (1.70–2.88)	1.52 (1.14–2.02)	1.42 (1.06–1.92)
**Hospitalization for heart failure**					
Young	97/1,580	Ref	Ref	Ref	Ref
Elderly/AF	127/943	2.30 (1.77–3.00)	2.32 (1.76–3.03)	1.51 (1.14–2.01)	1.47 (1.11–1.96)
Metabolic	113/1,037	1.83 (1.39–2.39)	1.81 (1.38–2.37)	1.46 (1.10–1.93)	1.46 (1.10–1.93)
Ischemic	125/1,122	1.83 (1.40–2.39)	1.77 (1.35–2.31)	1.63 (1.23–2.16)	1.60 (1.20–2.12)
Lean diabetic	217/1,185	3.23 (2.54–4.11)	3.25 (2.56–4.13)	2.07 (1.60–2.68)	1.99 (1.52–2.60)

Model 1 adjusted for age and sex. Model 2 adjusted for model 1 variables plus geographic zone, previous hospitalization for HF, NYHA class, and HF type (heart failure with preserved ejection fraction or heart failure with reduced ejection fraction). Model 3 adjusted for model 2 variables plus beta-blockers, angiotensin-converting enzyme inhibitors/angiotensin receptor blockers, diuretics, and mineralocorticoid receptor antagonists.

AF, atrial fibrillation.

The type of HF (HFrEF or HFpEF) modified the associations of multimorbidity groups with the primary combined outcome (*P*_interaction_ = 0.008), such that in HFpEF, only the lean diabetic group was associated with a higher proportion of the primary combined outcome compared to the young group (HR 2.57, 95% CI 1.19–5.59) when adjusted for age, sex, geographic zone, previous hospitalization for HF, and NYHA class.

## Discussion

To the best of our knowledge this study provides the first prospective multinational data on multimorbidity patterns among Asian patients with HF. We found several interesting results. First, rather than occurring in isolation, comorbidities naturally clustered among Asian patients with HF and could be categorized into 5 distinct patterns: elderly/AF, metabolic, young, ischemic, and lean diabetic. Second, different patterns of multimorbidity were associated with different underlying patterns of cardiac remodeling. Third, striking geographic differences were observed in the distribution of multimorbidity groups across Asia. Fourth, and most importantly, multimorbidity groups were differentially associated with the prespecified primary combined outcome of all-cause mortality and HF-related hospitalization. These data highlight the importance of multimorbidity in patients with HF, improve our understanding of the role of multimorbidity in the pathophysiology of HF, and pave the way for a tailored approach to patients with HF.

Previous studies have identified subgroups in HF using cluster analyses [10,26–28]. Ahmad et al. reported one of the first applications of a cluster analysis to identify clinical phenotypes of patients with HFrEF in the HF-ACTION study [10]. Based on clinical characteristics including ECG data, biomarkers (NT-proBNP), and signs and symptoms of HF, the authors identified 4 groups: a young group with high BMI, an elderly group with high rates of comorbidities, an ischemic cardiomyopathy group, and a non-ischemic cardiomyopathy group [10]. However, this study included only patients with HFrEF, and only patients from a single clinical trial, which predominantly included white men with ischemic cardiomyopathy. Similar studies have been performed in HFpEF patients alone [28,29], with similar subgroup findings. Based on the selected variables, these prior studies have been postulated to classify patients predominantly based on HF severity, with differences in survival driven mainly by differences in age and NT-proBNP [30]. Another study, by Lee et al., investigated comorbidity profiles in hospitalized HF patients using ICD codes in a US nationwide database [31]. The authors found a lifestyle profile, with high rates of diabetes and obesity, a renal profile, with high rates of renal disease and hypertension, a neurovascular profile (hypertension plus cerebrovascular disease), and a common group (high rate of hypertension). The hypertensive (common group) patients comprised the highest proportion (47%) among patients hospitalized for HF in the US, while the renal patients comprised the second highest proportion (30%), followed by the lifestyle (20%) and neurovascular (4%) patients with HF. This study relied on data from the Nationwide Inpatient Sample (NIS) database, and the depth of investigation was limited by the quality and detail of data collected [31].

Our study extends the prior literature by providing data on multimorbidity patterns and their echocardiographic correlates and association with QoL, mortality, and hospitalization for HF, in a large, well-characterized multinational Asian cohort of patients with HFrEF and HFpEF. We found novel multimorbidity patterns unique to Asia such as the lean diabetic group. Most noteworthy in our study was the prominence of the lean diabetic group in Southeast Asia (particularly Malaysia and Singapore). This was surprising given the rise in obesity in this zone [32]. Southeast Asia is home to a rapidly growing population of >600 million people, and is notable for its rapid epidemiological transition from “the age of receding pandemics” to “the age of degenerative and man-made disease” and now “the age of declining cerebrovascular mortality, ageing, lifestyle modifications, and resurgent diseases” [33,34] within the generation of adults now presenting with HF. The thrifty gene hypothesis [35] may explain the extraordinarily high rates of diabetes as a risk factor for HF in spite of the absence of overt obesity. Indeed, previous studies have shown that the prevalence of diabetes among Asian individuals is far greater than among white individuals and that diabetes occurs on average at a far lower BMI [36]. Furthermore, diabetes is associated with higher rates of mortality and hospitalization for HF in Asian patients with HF than in white patients with HF. Here we showed that among Asian patients with HF, the lean diabetic phenotype was associated with the highest rates of the primary combined outcome, with more than twice as many deaths or hospitalizations for HF compared to the young group. This is potentially driven by the high proportion of CKD in these patients, which is a strong determinant of mortality and hospitalizations [37]. Of note, the lean diabetic patients experienced higher rates of the primary combined outcome compared to obese diabetic patients in the metabolic group.

In Asia, the healthcare topography in terms of government health expenditure, availability of universal health insurance coverage, and reliance on private payment varies greatly, and this may contribute to disparities in care across the region. For instance, we have previously shown that there was enormous variation in utilization of implantable cardiac defibrillators in eligible patients in our cohort, which was associated with geographic variations in out-of-pocket health expenditure and total government health expenditure [15]. The extent to which these factors may have contributed to the regional differences in multimorbidity phenotypes and differences in all-cause mortality and hospitalization for HF warrants further study. Given that genetic background may be determined by ethnicity [38], future studies are warranted to determine possible genetic factors underlying the predominance of particular multimorbidity groups in different ethnicities.

Comorbidities are associated with certain pattern of cardiac structural and functional changes in HF [39]. Previous studies have shown that single comorbidities such as CKD, diabetes, and obesity affect cardiac structure and function both in patients with HF and in the general population [39–42]. Furthermore, a greater burden (number) of comorbidities is associated with indices of cardiac mechanics [43]. However, prior studies did not examine the cumulative effect of specific combinations of comorbidities. The prospective design of our study, with standardized echocardiography by protocol, enabled our detailed interrogation into cardiac structural and functional changes that potentially underlie the different clinical behaviors of patient groups. We found an expected association between the metabolic group and HFpEF, as well as between the ischemic group and HFrEF. More surprising was the association of the lean diabetic group with the greatest extent of concentric remodeling, LVH, and diastolic dysfunction, even more so than in the obese diabetic metabolic group in HFpEF, thus offering a potential explanation for the higher rates of the primary combined outcome seen in the lean diabetic group. Importantly, this provides clinical evidence of cardiometabolic disturbance as a key driver of cardiac dysfunction, apart from the confounding influence of weight gain per se, and supports the recent development of drugs targeting cardiometabolic pathways in HF [44]. In fact, our data suggest that these cardiometabolic agents may have unique application in specific Asian populations, as opposed to weight loss as a therapeutic strategy in Western populations [45]. Surprising was the association of the young group with the greatest prevalence of eccentric hypertrophy, even more so than the ischemic group in HFrEF, and despite the relative youth and strikingly low prevalence of comorbidities of individuals in the young group.

Our findings carry implications for clinical surveillance and management of patients with HF in different regions of Asia, as well as for design of global clinical trials in HF. This study shows that comorbidities in patients with HF cluster into distinct multimorbidity groups that affect mortality and hospitalization for HF beyond the sum of their parts. Future studies should take the combinations of comorbidities into account, which could drive decisions in personalized patient care based on survival as well as time to hospitalization for HF. Furthermore, patients from Southeast Asia with diabetes, even in the absence of obesity, warrant surveillance for HFpEF, and trials targeting HFpEF may enrich their populations by including lean diabetic patients from the region.

### Strengths and limitations

We acknowledge potential bias in site selection and willingness of patients to participate in a prospective registry, particularly across a huge geography of 11 regions, with disparate healthcare systems at different stages of evolution [15]. Site selection in the ASIAN-HF registry was based on the size of the region, geographic location of the site within the region, patient population served, HF patient volume, and availability of expertise in echocardiography. Screening logs were encouraged but not available from all sites. Nevertheless, every effort was made to ensure protocol adherence and standardization, including language translations specific to each region, on-site investigator training, regular monitoring (both in person and remote), and centralized database management. The representativeness of the ASIAN-HF registry has been discussed previously [4]. There is a paucity of multinational data on patients with HF in Asia. Therefore, we can only rely on comparisons to single-center studies or studies reporting on only a few countries in Asia. Previous results have shown that data on patients in the ASIAN-HF registry are consistent with prior reports from single Asian nations [46–50]. This suggests that patients included in ASIAN-HF registry are representative of patients with HF in the region. Although our cohort was prospectively enrolled and followed up, we included prevalent HF cases and their risk factors at baseline, with the potential for survival bias and reverse causality. For instance, the fact that the highest risk of the primary combined outcome was in the lean diabetic group may have been because these patients were frailer or had lost weight in the months leading up to inclusion. Of note, baseline severity of HF as measured by NYHA class was similar between the ischemic and metabolic groups. Nonetheless, while every effort has been made to correct for potential confounders in survival analyses, some unmeasured factors might have influenced differences in survival between groups. Particular strengths of this study include the prospective design, uniform comprehensive data collection, detailed echocardiographic characterization, and close follow-up with independent adjudication of outcomes. We also used state-of-the-art statistical methods: LCAs are hypothesis generating and provide us with potential new insights into multimorbidity profiles of patients with HF.

### Conclusion

These first prospective multinational data on multimorbidity patterns among Asian patients with HF showed that comorbidities naturally clustered in 5 distinct groups: elderly/AF, metabolic, young, ischemic, and lean diabetic. Different multimorbidity groups were associated with different underlying patterns of cardiac remodeling, and were differentially related to the primary combined outcome of all-cause mortality and hospitalization for HF, as well as to the secondary outcomes of all-cause mortality alone and hospitalization for HF alone. Striking geographic differences were observed in the distribution of multimorbidity groups across Asia. These data underscore the importance of multimorbidity in patients with HF and the need for more comprehensive approaches in phenotyping patients with HF and multimorbidity.

## Supporting information

S1 Checklist STROBE checklist. (DOCX)

S1 Table Regions included in the ASIAN-HF registry with enrollment number and income level. (XLSX)

S2 Table BIC values per number of classes. (XLSX)

S3 Table Probabilities per comorbidity for multimorbidity group membership. (XLSX)

S1 Text ASIAN-HF executive committee and participating institutions. (DOCX)

## Abbreviations

ACEi: angiotensin-converting enzyme inhibitor
AF: atrial fibrillation
ARB: angiotensin receptor blocker
ASIAN-HF: Asian Sudden Cardiac Death in Heart Failure
BIC: Bayesian information criterion
BMI: body mass index
BSA: body surface area
CAD: coronary artery disease
CKD: chronic kidney disease
COPD: chronic obstructive pulmonary disease
ECG: electrocardiography
eGFR: estimated glomerular filtration rate
HF: heart failure
HFpEF: heart failure with preserved ejection fraction
HFrEF: heart failure with reduced ejection fraction
HR: hazard ratio
KCCQ: Kansas City Cardiomyopathy Questionnaire
LCA: latent class analysis
LV: left ventricular
LVEF: left ventricular ejection fraction
LVH: left ventricular hypertrophy
LVM: left ventricular mass
MRA: mineralocorticoid receptor antagonist
NYHA: New York Heart Association
OR: odds ratio
PAVD: peripheral arterial and venous disease
QoL: quality of life
RWT: relative wall thickness
WHO: World Health Organization
