# Correction: Multimorbidity in patients with heart failure from 11 Asian regions: A prospective cohort study using the ASIAN-HF registry

**DOI:** 10.1371/journal.pmed.1002583

**Published:** 2018-05-25

**Authors:** Jasper Tromp, Wan Ting Tay, Wouter Ouwerkerk, Tiew-Hwa Katherine Teng, Jonathan Yap, Michael R. MacDonald, Kirsten Leineweber, John J. V. McMurray, Michael R. Zile, Inder S. Anand, A. Mark Richards, Carolyn S. P. Lam, ASIAN-HF authors

Prof. A. Mark Richards is not included in the author byline. He should be listed as the eleventh author and affiliated with Cardiovascular Research Institute, National University Heart Centre, Singapore, Singapore; National University Health System, Singapore, Singapore; National University of Singapore, Singapore, Singapore; Christchurch Heart Institute, University of Otago, Christchurch, New Zealand. The contributions of this author are as follows: Conceptualization, Funding Acquisition, Investigation, Project Administration, Resources, Supervision, Writing–Original Draft Preparation.
