# Supplementary material for: Multimorbidity in patients with heart failure from 11 Asian regions: A prospective cohort study using the ASIAN-HF registry
Source: PLoS Med. 2018 Mar 27;15(3):e1002541. doi: 10.1371/journal.pmed.1002541 (PMC5870945; doi:10.1371/journal.pmed.1002541)
Supplement: S1 Text — (DOCX) [file pmed.1002541.s005.docx]

**THE ASIAN-HF EXECUTIVE COMMITTEE**

- Professor A. Mark Richards (as Chairman), Cardiovascular Research Institute, National University of Singapore, Singapore. Email: [mdcarthu@nus.edu.sg](mailto:mdcarthu@nus.edu.sg)
- Professor Carolyn S.P. Lam (as Principal Investigator), National Heart Centre Singapore, Duke-NUS Medical School, Singapore. Email: [carolyn.lam@duke-nus.edu.sg](mailto:carolyn.lam@duke-nus.edu.sg)
- Professor Inder Anand (as Director, Publications Committee), University of Minnesota Medical School, VA Medical Center Minneapolis and San Diego, United States of America. Email: [anand001@umn.edu](mailto:anand001@umn.edu)
- Dr Chung-Lieh Hung, Mackay Memorial Hospital, Taipei, Taiwan. Email: [jotaro3791@gmail.com](mailto:jotaro3791@gmail.com)
- Professor Lieng Hsi Ling (as Director, Echo Core Laboratory), Cardiovascular Research Institute, National University of Singapore, Singapore. Email: [lieng_hsi_ling@nuhs.edu.sg](mailto:lieng_hsi_ling@nuhs.edu.sg)
- Dr Houng Bang Liew, Queen Elizabeth II Hospital, Clinical Research Center, Sabah, Malaysia. Email: [hbliew22@gmail.com](mailto:hbliew22@gmail.com)
- Dr Calambur Narasimhan, Care Hospital, Hyderabad, India. Email: [calambur@hotmail.com](mailto:calambur@hotmail.com)
- Dr Tachapong Ngarmukos, Ramathibodi Hospital, Mahidol University, Bangkok, Thailand. Email: [tachaponis.nga@mahidol.ac.th](mailto:tachaponis.nga@mahidol.ac.th)
- Dr Sang Weon Park, SeJong General Hospital, Seoul, South Korea. Email: [swparkmd@gmail.com](mailto:swparkmd@gmail.com)
- Dr Eugenio Reyes, Manila Doctors Hospital, Manila, Philippines. Email: [eugenereyes@yahoo.com](mailto:eugenereyes@yahoo.com)
- Professor Bambang B. Siswanto, National Cardiovascular Center Universitas Indonesia, Jakarta, Indonesia. Email: [bambbs@gmail.com](mailto:bambbs@gmail.com)
- Professor Wataru Shimizu, Department of Cardiovascular Medicine, Nippon Medical School, Tokyo, Japan. Email: [wshimizu@nms.ac.jp](mailto:wshimizu@nms.ac.jp)
- Professor Shu Zhang, Fuwai Cardiovascular Hospital, Beijing, People’s Republic of China. Email: [zsfuwai@vip.163.com](mailto:zsfuwai@vip.163.com)

**COUNTRY AND SITE INVESTIGATORS**

China

Fuwai Hospital: **Shu Zhang** (Country PI), Xiaohan Fan, Keping Chen. Ruijin Hospital, Shanghai Jiaotong university: Liqun Wu, Yucai Xie, Qi Jin, Tianyou Ling. The First Affiliated Hospital With Nanjing Medical University: Xinli Li, Fang Zhou, Yanli Zhou, Dongjie Xu, Haifeng Zhang. Zhongshan Hospital Fudan University: Yangang Su, Xueying Chen, Shengmei Qin, Jingfeng Wang, Xue Gong, Zhaodi Wu.

Hong Kong

The Chinese University of Hong Kong: **Cheuk Man Yu** (Country PI).

India

CARE Hospital: **Calambur Narasimhan** (Country PI), B K S Sastry, Arun Gopi, K Raghu, C Sridevi, Daljeet Kaur. Care Institute of Medical Sciences: Ajay Naik, Keyur Parikh, Anish Chandarana, Urmil Shah, Milan Chag, Hemang Baxi, Satya Gupta, Jyoti Bhatia, Vaishali Khakhkhar, Vineet Sankhla, Tejas Patel, Vipul Kapoor. Hero Dayanand Medical College Heart Institute: Gurpreet Singh Wander, Rohit Tandon. Medanta-The Medicity: Vijay Chopra, Manoj Kumar, Hatinder Jeet Singh Sethi, Rashmi Verma, Sanjay Mittal. Sir Ganga Ram Hospital: Jitendra Sawhney, Manish Kr. Sharma. Westfort Hi-Tech Hospital Ltd: Mohanan Padinhare Purayil.

Indonesia

Rumah Sakit Jantung dan Pembuluh Darah Harapan Kita: **Bambang Budi Siswanto** (Country PI). RS Dr Hasan Sadikin: Pintoko Tedjokusumo, Erwan Martanto, Erwinanto. R S Khusus Jantung Binawaluya: Muhammad Munawar, Jimmy Agung Pambudi. RS Siloam Karawaci: Antonia Lukito, Ingrid Pardede, Alvin Thengker, Vito Damay, Siska Suridanda Danny, Rarsari Surarso.

Japan

Nippon Medical School: **Wataru Shimizu** (Country PI), Takashi Noda, Ikutaro Nakajima, Mitsuru Wada, Kohei Ishibashi. Kinki University Hospital Cardiovascular Center: Takashi Kurita, Ryoubun Yasuoka. Nippon Medical School Hospital: Kuniya Asai, Kohji Murai, Yoshiaki Kubota, Yuki Izumi.Toho University Omori Medical Center: Takanori Ikeda, Shinji Hisatake, Takayuki Kabuki, Shunsuke Kiuchi, Tokyo Women's Medical University: Nobuhisa Hagiwara, Atsushi Suzuki, Dr. Tsuyoshi Suzuki.

Korea

SeJong General Hospital: **Sang-Weon Park** (Country PI), Suk Keun Hong, SookJin Lee, Lim Dal Soo, Dong-Hyeok Kim. Korea University Anam Hospital: Jaemin Shim, Seong-Mi Park, Seung-Young Roh, Young Hoon Kim, Mina Kim, Jong-Il Choi. Korea University Guro Hospital: Jin Oh Na, Seung Woon Rha, Hong Seog Seo, Dong Joo Oh, Chang Gyu Park, Eung Ju Kim, Sunki Lee,

Severance Hospital, Yonsei University Health System: Boyoung Joung, Jae-Sun Uhm, Moon Hyoung Lee, In-Jeong Cho, Hui-Nam Park. Chonnam National University Hospital: Hyung-Wook Park, Jeong-Gwan Cho, Namsik Yoon, KiHong Lee, Kye Hun Kim. Korea University Ansan Hospital: Seong Hwan Kim.

Malaysia

Hospital Queen Elizabeth II: **Houng Bang Liew** (Country PI), Sahrin Saharudin, Boon Cong Beh, Yu Wei Lee, Chia How Yen, Mohd Khairi Othman, Amie-Anne Augustine, Mohd Hariz Mohd Asnawi, Roberto Angelo Mojolou, You Zhuan Tan, Aida Nurbaini Arbain, Chii Koh Wong. Institut Jantung Negara: Razali Omar, Azmee Mohd Ghazi, Surinder Kaur Khelae, David S.P. Chew, Lok Bin Yap, Azlan Hussin, Zulkeflee Muhammad, Mohd. Ghazi Azmee. University Malaya Medical Centre: Imran Zainal Abidin, Ahmad Syadi Bin Mahmood Zhudi, Nor Ashikin Md Sari, Ganiga Srinivasaiah Sridhar, Ahmad Syadi Mahmood Zuhdi. Muhammad Dzafir Ismail. Sarawak General Hospital Heart Centre: Tiong Kiam Ong, Yee Ling Cham, Ning Zan Khiew, Asri Bin Said, Alan Yean Yip Fong, Nor Hanim Mohd Amin, Keong Chua Seng, Sian Kong Tan, Kuan Leong Yew.

Philippines

Manila Doctors Hospital: **Eugenio Reyes** (Country PI), Jones Santos, Allan Lim. Makati Medical Center: Raul Lapitan, Ryan Andal, Philippine Heart Center: Eleanor Lopez.

Singapore

National Heart Centre Singapore: **Carolyn S.P. Lam** (Country PI), Kheng Leng David Sim, Boon Yew Tan, Choon Pin Lim, Louis L.Y. Teo, Laura L.H. Chan. National University Heart Centre: Lieng Hsi Ling, Ping Chai, Ching Chiew Raymond Wong, Kian Keong Poh, Tan Tock Seng Hospital: Poh Shuan Daniel Yeo, Evelyn M. Lee, Seet Yong Loh, Min Er Ching, Deanna Z.L. Khoo, Min Sen Yew, Wenjie Huang. Changi General Hospital-Parent: Kui Toh Gerard Leong, Jia Hao Jason See, Yaozong Benji Lim, Svenszeat Tan, Colin Yeo, Siang Chew Chai. Singapore General Hospital-Parent: Fazlur Rehman Jaufeerally, Haresh Tulsidas, Than Aung. Khoo Teck Puat Hospital: Hean Yee Ong, Lee Fong Ling, Dinna Kar Nee Soon

Taiwan

Mackay Memorial Hospital, Taipei, Taiwan: **Chung-Lieh Hung** (Country PI), Hung-I Yeh,Jen-Yuan Kuo, Chih-Hsuan Yen. National Taiwan University Hospital: Juey-Jen Hwang, Kuo-Liong Chien, Ta-Chen Su, Lian-Yu Lin, Jyh-Ming Juang, Yen-Hung Lin, Fu-Tien Chiang, Jiunn-Lee Lin, Yi-Lwun Ho, Chii-Ming Lee, Po-Chih Lin, Chi-Sheng Hung, Sheng-Nan Chang, Jou-Wei Lin, Chih-Neng Hsu. Taipei Veterans General Hospital: Wen-Chung Yu, Tze-Fan Chao, Shih-Hsien Sung, Kang-Ling Wang, Hsin-Bang Leu, Yenn-Jiang Lin, Shih-Lin Chang, Po-Hsun Huang, Li-Wei Lo, Cheng-Hsueh Wu. China Medical University Hospital: Hsin-Yueh Liang, Shih-Sheng Chang, Lien-Cheng Hsiao, Yu-Chen Wang, Chiung-Ray Lu, Hung-Pin Wu, Yen-Nien Lin, Ke-Wei Chen, Ping-Han Lo, Chung-Ho Hsu, Li-Chuan Hsieh.

Thailand

Ramathibodi Hospital: **Tachapong Ngarmukos** (Country PI), Mann Chandavimol, Teerapat Yingchoncharoen, Prasart Laothavorn. Phramongkutklao Hospital:Waraporn Tiyanon. Maharaj Nakor
